# Arterial Hypertension as a Risk Comorbidity Associated with COVID-19 Pathology

**DOI:** 10.1155/2020/8019360

**Published:** 2020-12-04

**Authors:** Alexander Kamyshnyi, Inna Krynytska, Victoriya Matskevych, Mariya Marushchak, Oleh Lushchak

**Affiliations:** ^1^Department of Microbiology, Virology and Immunology, I. Horbachevsky Ternopil National Medical University, Ternopil, Ukraine; ^2^Department of Functional and Laboratory Diagnostics, I. Horbachevsky Ternopil National Medical University, Ternopil, Ukraine; ^3^Department of Radiology and Radiation Medicine, Ivano-Frankivsk National Medical University, Ivano-Frankivsk, Ukraine; ^4^Department of Biochemistry and Biotechnology, Vasyl Stefanyk Precarpathian National University, Ivano-Frankivsk, Ukraine

## Abstract

Coronavirus disease 2019 (COVID-19), caused by the novel coronavirus severe acute respiratory syndrome-coronavirus-2 (SARS-CoV-2), is an ongoing global public health challenge. Current clinical data suggest that, in COVID-19 patients, arterial hypertension (AH) is one of the most common cardiovascular comorbidities; it can worsen outcomes and increase the risk of admission to intensive care unit (ICU). The exact mechanisms through which AH contributes to the poor prognosis in COVID-19 are not yet clear. The putative relationship between AH and COVID-19 may be linked to the role of angiotensin-converting enzyme 2 (ACE2), a key element of the AH pathophysiology. Another mechanism connecting AH and COVID-19 is the dysregulation of the immune system resulting in a cytokine storm, mediated by an imbalanced response of T helper cells subtypes. Therefore, it is essential to optimize blood pressure control in hypertensive patients and monitor them carefully for cardiovascular and other complications for the duration of COVID-19 infection. The question whether AH-linked ACE2 gene polymorphisms increase the risk and/or worsen the course of SARS-CoV-2 infection should also receive further consideration.

## 1. Introduction

The emergence of a new zoonotic infection, severe acute respiratory syndrome-coronavirus-2 (SARS-CoV-2), has created an extraordinary challenge for the healthcare systems globally [1]. This single-stranded RNA virus is the seventh described human coronavirus. While it differs from the other coronaviruses known to cause the common cold (229E, OC43, NL63, and HKU1), it is similar to the recently emerging zoonotic severe acute respiratory syndrome coronavirus (SARS-CoV), the cause of SARS outbreak in 2002, and the Middle East respiratory syndrome coronavirus (MERS-CoV), causing MERS, which emerged in 2012. Since SARS-CoV-2 shares 89–96% nucleotide sequence with bat coronaviruses, it is likely to have originated in bats, an emergence path similar to other coronaviruses [2].

The virus possesses relatively low virulence and high contagiousness and can be transmitted even during asymptomatic phase. These factors contribute to the rapid spread of SARS-CoV-2 across the globe, causing a pandemic [1]. The first case of coronavirus disease 2019 (COVID-2019) was recorded in the Hubei province of China on December 8, 2019 [3]. By September 11, 2020, WHO reported 28,351,973 cases of COVID-19 resulting in 914,256 deaths. By the same date, in Ukraine, according to the National Public Health Center, the number of confirmed cases of COVID-19 has reached 148,756, and 3,076 of them were lethal. Some clinicians and scientists predict that up to 80% of the human population can become infected by SARS-CoV-2. The outcomes and long-term consequences of an infection considerably vary in every individual. In this review, we highlighted the mechanisms of immune response to SARS-CoV-2, a factor that can increase the risk of hospitalization and mortality under infection. We also summarized clinical data on the role of hypertension as an important risk factor for SARS-CoV-2-induced pathologies.

## 2. Immune Response to SARS-CoV-2

**Figure 1:**
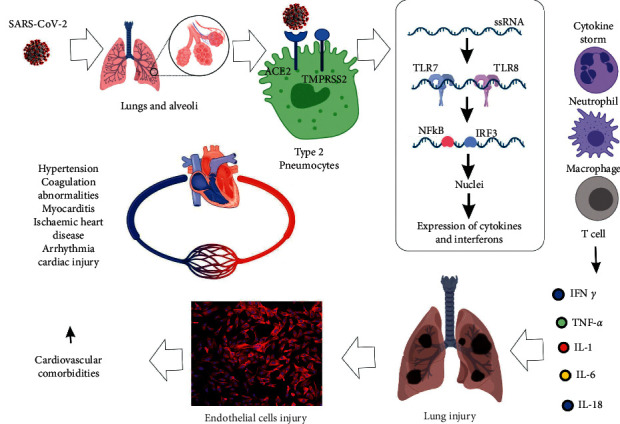
Progression of SARS-CoV-2 infection and the mechanisms implicated in the development of cardiovascular comorbidities.

The complete mechanism of immune response to COVID-19 infection caused by SARS-CoV2 is still under investigation. However, similar to most coronaviruses, the SARS-CoV2 utilizes its structural proteins to gain entry into the host cell cytosol, as well as suppress signaling pathways, particularly those with the toll-like receptors (TLRs) (Figure 1) [4]. This interaction initiates a signal cascade involving the transcriptional factors IRF3 and NF-kB. These factors are further translocated into the nucleus to activate expression of proinflammatory cytokines and interferons (IFNs), particularly type 1 IFN (IFN1).

The magnitude of immune system response to viral infection is strongly mediated by IFN1 [5]. It can both inhibit viral replication and induce the effective adaptive immune response. Angiotensin-converting enzyme 2 (ACE2), the entry receptor to SARS-CoV-2, is extensively expressed in the Type 2 pneumocytes of the lungs [6]. SARS-CoV-2 also infects macrophages and T cells in a key contributor of pathogenesis progression [7]. However, it is still unclear if the virus infects all immune cells, since only a percentage of lung monocytes/macrophages express ACE2 [6]. It is possible that other receptors are involved in the infection of the immune cells with low levels of ACE2 expression. Alternatively, the antibody-dependent enhancement may be the mechanism mediating an alternate entry mode.

Accurate antiviral response requires that pathogen-associated molecular patterns (PAMPs) are recognized by innate immune cells. Cytosolic RNA sensors RIG-I/MDA5 and endosomal RNA receptors on TLR7 and TLR8 detect viral genomic RNA or its replication intermediates, including dsRNA Activation of downstream signaling cascade induces nuclear translocation of NF-*κ*B and IRF3 and expression of type I IFNs and other proinflammatory cytokines. These initial responses form the first line of defense against viral infection at the entry point [8, 9]. Additionally, IFN1 activates the JAK-STAT pathway via IFNA receptor inducing STAT1 and STAT2 phosphorylation by JAK1 and TYK2, respectively. Effective suppression of viral replication is achieved by activated forms of STATs interacting with IRF9; the resulting complex translocates to the nucleus, where it induces transcription of IFN-stimulated genes (ISGs) via IFN-stimulated response element [8, 10].

A retrospective analysis of the clinical data of patients with viral pneumonia by Guo et al. showed that, in the deceased group, the absolute count levels of CD3^+^ T cells, CD3^+^ CD8^+^ T cells, and CD3^+^ CD4^+^ T cells were significantly lower compared with the survival group, suggesting that inflammatory factor levels in the deceased group were also higher than those in the survival group [11].

In recent years, the response of T cells to SARS-CoV infection was extensively studied. An analysis of 128 convalescent samples showed that CD8^+^ T cells had a more frequent response with higher magnitude compared to CD4^+^ T cells [12]. In patients with severe complications, polyfunctional CD8^+^ T cells and CD4^+^ T cells comprised significantly higher proportion of T cells compared with the samples from patients with mild-to-moderate complications. Robust T cell responses were associated with higher titres of neutralizing antibodies. In contrast, the diseased group samples presented elevated levels of IL4, IL5, and IL10. In about 70% of the cases, the response was directed against the spike, membrane, capsid, or envelope structural proteins. The early increase in CD8^+^ T cell population was also found to correlate with severity of disease caused by MERS-CoV infection, while Th1 type helper T cells present in recovery phase [13]. Airway memory CD4^+^ T cells specific for conserved epitope for SARS-CoV and MERS-CoV were shown to protect against lethality [14]. Moreover, it is still unclear whether Th17 play the protective or harmful role during coronavirus infection, since neutrophils are known to play a detrimental role in all infections [5].

## 3. Comorbidities Associated with Increased Risks of COVID-19-Induced Pathologies

Respiratory symptoms similar to a mild flu-like illness are the main clinical sign of COVID-19. These symptoms, however, can exacerbate into acute respiratory distress syndrome (ARDS) or fulminant pneumonia, both potentially lethal conditions. Certain comorbidities are associated with increased risk of infection as well as poorer outcomes and mortality due to increased severity of lung injury. The most commonly reported comorbidities were arterial hypertension (AH, 30%), diabetes mellitus (DM, 19%), and coronary heart disease (CHD, 8%) [15]. In a clinical signs report on 41 patients, Huang et al. noted that 13 patients (32%) had underlying diseases, including cardiovascular diseases (CVDs), DM, AH, and chronic obstructive pulmonary disease (COPD) [16]. In another group of 191 patients from Wuhan province (China), any of the comorbidities listed previously presented in 48% of all cases, with AH presenting in 30% of the patients and CVD in 8% [17]. However, about 67% of the patients who died had comorbidities, including 48% with AH and 13% with diagnosed CVD. Another study of138 COVID-19 cases showed that 64 patients (46.4%) had comorbidities [18]. Moreover, patients admitted to the intensive care unit (ICU) had a higher proportion of comorbidities (72.2%) compared with those who did not require ICU admission (37.3%). This suggests that comorbidities are risk factors for poor outcomes. An analysis of 1,099 patients (both outpatients and inpatients) with COVID-19 showed that 24% had a comorbidity; this proportion rose to 58% among cases requiting intubation or resulting in death. About 15% had AH (36% among those requiring intubation or resulting in death) and 2.5% had CHD (9% among those requiring intubation or resulting in death) [19]. Menter and coauthors analyzed autopsy results of 21 COVID-19 patients hospitalized at the University Hospital Basel and Cantonal Hospital Baselland (Switzerland) and determined that the primary cause of death was respiratory failure associated with exudative diffuse alveolar damage and massive capillary congestion, often accompanied by microthrombi even though the patients might have received anticoagulant treatment. Most of the patients suffered from one or more comorbidities such as AH, obesity, CVD, or DM [20].

Preexisting CVDs increase susceptibility to COVID-19, similar to any other comorbidity. What is more, COVID-19 can aggravate underlying CVDs and even result in emergence of cardiac complications [1]. CVD was also found to be a frequent comorbidity in SARS and MERS patients, reaching, in the former case, 8%. The prevalence of DM in SARS patients was 11%, and additional comorbidities increased the risk of death twelvefold [21]. About 50% of the patients diagnosed with MERS had DM and AH, while CVD was present in approximately 30% of patients [22]. COVID-19 patients, especially those with a more severe disease, also present with an increased incidence of cardiovascular comorbidities.

Cardiovascular complications in COVID-19 involve mechanisms such as direct myocardial injury, systemic inflammation, plaque rupture, and coronary thrombosis [1, 17, 23, 24]. Available data supports that the number of CVDs in infected patients correlates with mortality [25]. The overall case fatality rate (CFR) was 2.3% in the entire cohort but significantly higher in patients with AH, DM, and CVD (6%, 7.3%, and 10.5%, respectively). The Chinese Center for Disease Control (CDC) has reviewed 72,314 confirmed, suspected, and asymptomatic COVID-19 cases to conclude that CFR was 0.9% in the patients with no comorbid medical conditions. The majority of patients among the confirmed cases were 30–79 years old (86.6%), and preexisting diseases increased the risk of COVID-19 mortality. The death rate increased to 10.5% in patients with CVD, 7.3% in diabetic patients, 6.3% in patients with chronic respiratory disease, 6.0% in patients with AH, and 5.6% in patients with cancer [26]. Notably, data from different regions of the world suggest dissimilar impact of various cardiovascular comorbidities on the clinical outcomes. Some EU countries reported higher CFRs compared with China or countries of other regions [1].

Of all CVDs, AH is the single largest contributor to disability-adjusted life years lost [27]. Worldwide figures suggest that, in 2010, 1.39 billion (31.1%) adults had hypertension. Comparatively higher prevalence of AH among adults was observed in low-to middle-income countries (31.5%, or 1.04 billion people) than in high-income countries (28.5%, or 349 million people) [28]. In the over-60 age cohort, the majority had AH and thus appeared to be at a higher risk when infected with SARS-CoV-2. However, it is still unclear if AH significantly increases susceptibility to this virus. Current data indicate that 15–40% of the cases are associated with high blood pressure [29]. A recent Chinese study shows that the prevalence of AH in the cohort with a less severe form of COVID-19 (13.4%) was lower than in those with severe form of the disease (23.7%). However, that study presented a complex outcome showing higher predominance of AH in subjects with a poor composite outcome (35.8% compared with 13.7%) [19]. While AH was diagnosed in 12.8% of the entire study group, it was found in 39.7% of the patients who died [26]. Another study showed that, in patients with COVID-19, AH increased the odds ratio for mortality by 3.05 (95% CI 1.57–5.92) [28]. Zuin et al. also found that hypertensive patients with COVID-19 also had a significantly higher risk of mortality compared with normotensive patients (OR 3.36, 95% CI 1.96–5.74) [30].

In the aged population, a more severe course of COVID-19 and higher mortality rate might be explained by the higher prevalence of AH in this cohort [19]. Data collected by the National Health Commission of China show that 35% of the patients diagnosed with COVID-19 had AH [31]. Lastly, a recent meta-analysis of eight studies from China which included 46,248 infected patients revealed that AH was the most prevalent comorbidity (17 ± 7%, 95% CI 14–22%) [32]. Additionally, a meta-analysis of 1,527 Chinese COVID-19 patients found the presence of DM in 9.7% of the cases, cardiocerebrovascular disease in 16.4% cases, and AH in 17.1% cases [24]. Cardiocerebrovascular disease, AH, and DM were associated with the increased risk of severe disease or the need of ICU admission, supporting prognostic value of these comorbidities [1, 3].

## 4. Arterial Hypertension and COVID-19

The prevalence of AH in COVID-19 patients ranged, in different studies, from 15% to 20% [16, 19] and from 30% to 35% [17, 33]. The cohort of the patients with increased prevalence of AH also had significantly higher average age, which suggests that age is the most important reason for the difference in the proportion of hypertensive COVID-19 patients among the studies. Advanced age was associated with higher prevalence of other comorbidities as well, such as diabetes, renal deficiency, AH, and obesity that occur in a large proportion of the hypertensive population [34].

SARS-CoV-2 infection is mediated by binding of the viral surface spike protein to the human ACE2 receptor, following activation of the spike protein by a transmembrane protease serine 2 (TMPRSS2) [31, 35]. The association between SARS-CoV-2 and ACE2 points out to the involvement of AH in COVID-19 pathogenesis. AH could either play a direct role as a salient clinical predictor of disease severity or be a contributing factor to the deterioration late in the disease course, which is characterized by ARDS and systemic inflammatory response syndrome and/or multiple organ failure [36]. ACE2 is a key element of the renin-angiotensin-aldosterone system (RAAS), a critical pathway involved AH pathophysiology [37, 38].

The *ACE2* gene spans 39.98 kb of genomic DNA and contains 18 exons. It is located at the Xp22 locus on human *X* chromosome [39]. An ACE2 polymorphism, first documented in Chinese population, with three variants (rs4240157, rs4646155, and rs4830542), is associated with AH [40]. It is possible that ACE2 polymorphisms can affect SARS-CoV-2 susceptibility and COVID-19 outcome by influencing blood pressure through the RAAS and possibly increasing lung and heart damage via oxidative stress triggered by angiotensin II [39]. A recent work by Cao et al. [41] characterized 32 variants of ACE2, including seven hotspot variants (Lys26Arg, Ile486Val, Ala627Val, Asn638Ser, Ser692Pro, Asn720Asp, and Leu731Ile/Phe) in different populations. This suggests that some individuals could have increased susceptibility to SARS-CoV-2 infection.

Men have higher levels of ACE2 expression in the lungs than women, and individuals in Asian populations possess higher ACE2 transcript levels than populations of Caucasian and African descent [42]. Since ACE2 is located on the *X* chromosome, the presence of alleles conferring resistance to SARS-CoV-2 is a suggested mechanism behind the apparent lower female mortality rate [43]. Clinical reports published to date indicate that males comprise between 66% and 75% of the most severe COVID-19 cases [39]. For instance, in the analysis of 1,099 patients with a laboratory-confirmed COVID-19 and acute respiratory disease diagnosis, Guan et al. found that the patient median age was 47 years and 41.9% of the patients were females [19].

ACE2 is expressed in Type 2 pneumocytes [44] and appears to be a predominant entry point for the virus. ACE2 is also expressed in the heart muscle tissue, where it counteracts the effects of angiotensin II produced under excessive activation of the RAAS caused by AH [45]. The crucial role of ACE2 in mediating SARS-CoV infection in the heart was confirmed in an experimental mouse model. Animals with pulmonary infection caused by the human SARS-CoV developed ACE2-dependent myocardial infection with a marked decrease in ACE2 expression [46]. In addition to the heart and lung, ACE2 is also expressed in the kidneys, intestinal epithelium, and vascular endothelium, pointing to the mechanism behind the multiorgan failure often seen in SARS-CoV-2 infection [2].

SARS-CoV-2 primarily attacks alveolar epithelial cells, producing respiratory symptoms. Patients with CVD usually exhibit more severe symptoms, likely because of the upregulated ACE2 expression compared with healthy individuals [31]. RAAS inhibitors can increase the ACE2 levels. Experimental studies confirmed the increase in ACE2 tissue levels as a compensatory mechanism following the inhibition of the RAAS through either ACE inhibitors (ACEIs) or angiotensin II receptor blockers (ARBs). This suggests that such medications can be harmful in SARS-CoV-2 patients [47]. However, there is no direct evidence that ACEIs or ARBs treatments affect ACE2 levels in human tissues. Currently, there are valid reasons to terminate ACEIs or ARBs treatments in patients at risk of COVID-19 [36]. Moreover, some RAAS inhibitors may exert a potentially protective influence shown in experimental models [48]. Thus, if angiotensin II contributes to the internalization and intracellular degradation of ACE2, its inhibitor losartan reduced this effect, suggesting that ARBs may provide protection against viral at cell entry point [47].

Immune system dysregulation is another mechanism linking AH and COVID-19 [29, 49]. Insufficiently controlled blood pressure can promote dysregulation of the immune system. In humans, high circulating lymphocyte counts are positively correlated with AH, while hypertensive patients exhibit CD8^+^ T cell dysfunction [50, 51]. These immunosenescent CD8^+^ T cells are incapable of tackling viral infections effectively and instead might contribute to excess cytokine production, increasing the risk of complications. Effective control of high blood pressure using ACEI or ARBs treatments can partially reverse the dysregulation of immune system in hypertension [29].

Cytokines directly regulate ACE2 levels [52]. The decrease in ACE2 levels can be caused directly by the viral infection and ensue immune and inflammatory responses in the infected tissue. ACE2 is expressed in macrophages [53]. Notably, ACE3 knockout in leukocytes induced adipose inflammation [54], suggesting its possible contribution to inflammatory response.

Cytokine storm has been implicated in COVID-19-related cardiac involvement and is mediated by a deregulated response from T helper cells subtypes [16, 17, 55], as well as hypoxia-induced increase in intracellular Ca^2+^, resulting in the apoptosis of cardiac myocytes [31]. Interaction between the viral spike proteins and ACE receptor ensures viral entry into the alveolar cells with the concomitant of structural disintegration. The initiated inflammatory response was due to the release of proinflammatory cytokines such as IL6 and TNF *α* [56].

Severe cases of COVID-19 have been associated with high levels of proinflammatory cytokines, including IL2, IL7, IL10, TNF *α* IP10, MCP1, GCSF, and MIP1A [5, 16]. These results support the fact that lymphopenia and cytokine storm play a major role in the pathogenesis of COVID-19. These findings suggest that lymphopenia and cytokine storm play a major role in the pathogenesis of COVID-19 and are consistent with the data on SARS and MERS [57]. Additional histological studies of autopsy or biopsy results of different organs are needed to better understand this progression of severe complications.

## 5. Conclusions and Perspectives

AH is one of the most common COVID-19 comorbidities, and, in general, patients with CVD have increased risk of severe progression of the disease and its complications. Because of the high global prevalence of AH, it is important to take into account the association of COVID-19 outcomes with AH and antihypertensive medications when developing specific preventative measures and AH management protocols for these patients. Such measures should include strict emphasis on preventing infection risks (i.e., maintaining spatial distance of at least 1 m, following social isolation guidance, and access to high-grade personal protection equipment, such as gloves, goggles, face shields, and respirators). Consistent monitoring and management of blood pressure can help avoid broad BP fluctuations, which are associated with a higher risk of developing targeted (i.e., lung) or multiple organ failure. Moreover, it is important to avoid unnecessary and unmonitored changes to antihypertensive therapy. The exact cellular mechanisms, through which CVDs, including AH, aggravate COVID-19 prognosis, are still being investigated. Another issuer requiring further studies is the genetic predisposition linking ACE2 polymorphisms associated with AH to an increased risk and/or severe course of SARS-CoV-2 infection. A number of questions require additional study: does preexisting AH increase the risk of SARS-CoV-2 infection and/or worsen the course of COVID-19? Is the degree of AH related to high expression levels of ACE2, the SARS-CoV-2 receptor, in the heart and blood vessel endothelium? How the imbalances of the immune system caused by COVID-19 increase the severity of AH? Can modulation of the immune response in COVID-19 patients reduce the severity of AH and hypertensive damage to the target organs? What are the optimal AH ranges and therapies that can ensure a protective effect against COVID-19 and improve its clinical outcomes?
